# Blood culture result profile and antimicrobial resistance pattern: a report from neonatal intensive care unit (NICU), Asella teaching and referral hospital, Asella, south East Ethiopia

**DOI:** 10.1186/s13756-019-0486-6

**Published:** 2019-02-20

**Authors:** Abebe Sorsa, Jonas Früh, Loraine Stötter, Sileshi Abdissa

**Affiliations:** 1 Arsi University Asella College of Health Science, Asella, Ethiopia; 2 Hirsch-Institute of Tropical Medicine, Asella, Ethiopia

**Keywords:** Bacteria isolates, Neonatal sepsis, Antimicrobial resistance

## Abstract

**Background:**

Antimicrobial resistance is one of the major public health emergencies worldwide, and this trend didn’t spare developing countries like Ethiopia. The objective of this study was to evaluate patterns of bacterial isolates and local antimicrobial susceptibility patterns in neonatal sepsis.

**Methods:**

A hospital based observational study was conducted from April 2016 to May 2017 in Asella teaching and referral hospital (ATRH). A total of 303 neonates with clinical sepsis were included. Collected data were entered into EPI-INFO version 3.5.1 for cleanup; and then exported to SPSS version 21 for further analysis. Frequencies and proportion were used to describe the study population in relation to relevant variables.

**Results:**

Bacterial growth was detected in 88 (29.4%) of blood cultures. Predominantly isolated bacteria were coagulase negative *staphylococci* (CoNS) 22 (25%), *Escherichia coli* (E.Coli) 18 (20.5%) and *Staphylococcus* aureus 16 (18%). Resistance rates of S. aureus and CoNS against Ampicillin were 11 (69%) and 20 (91%) respectively. The resistance rate of *E. coli* against Ampicillin and Gentamycin were 12 (66.7%) and 10 (55.6%) while *Klebsiella spp.* resistance rate gets much higher against these two first line antibiotics [10 (91%) and 9 (82%) respectively]. Similarly, both Gram-positive and Gram-negative bacteria isolates were also highly resistant to third generation Cephalosporins, and 63 (72%) isolated bacteria showed multidrug-resistance. However; Gram-positive bacteria isolates had better susceptibility patterns to third line antibiotics like Clindamycin, Vancomycin and Ciprofloxacin while Gram-negative isolates had a higher susceptibility to Ciprofloxacin and Amikacin.

**Conclusion:**

CoNS, *S. aureus*, *E. coli* and *Klebsiella spp.* were the leading bacterial causes of neonatal sepsis in our study. They were highly resistant to first- and second-line empiric antimicrobial treatment used at NICU (Neonatal intensive care unit), reducing the antimicrobial choices for management of neonatal sepsis. Fortunately, the mentioned isolated bacteria remained susceptible to third line antibiotics used to treat neonatal sepsis.

## Background

Neonatal mortality contributes significantly to the infant mortality rates in developing countries, various conditions are responsible for neonatal mortality among which neonatal sepsis accounts for variable figures ranging from 26 to 50% [1–4]. Neonatal sepsis is defined as any sepsis diagnosed during the first 28 days of life and further sub classified as early onset neonatal sepsis if signs and symptoms of sepsis appeared within the first six days of life and classified as late onset sepsis if clinical features of sepsis are presented between 7 and 28 days of age [5]. Neonatal sepsis is mainly caused by different Gram-positive and Gram-negative bacteria and few cases by fungi like candida species. There is a significant geographical diversity of bacteria causing neonatal sepsis and the spectrum is constantly changing over time, even in the same place [5–7]. Antibiotic resistance has become a global threat. Reports of multidrug-resistant bacteria causing neonatal sepsis in developing countries are increasing, particularly in intensive care units. The clinical signs and symptoms of neonatal sepsis are subtle and nonspecific, making early diagnosis difficult and leading to high rate of empiric antibiotic utilization which could contribute for the selection and spread of antimicrobial resistant strains of bacteria. Knowing the causative agents of neonatal sepsis and their antimicrobial sensitivity patterns could enable to choose appropriate therapy for neonatal sepsis. Targeted antibiotic therapy plays a significant role in reduction of antimicrobial resistance [5, 6].

The objective of this study was to assess the etiology of neonatal sepsis and to provide local antimicrobial susceptibility patterns in the neonatal intensive care unit.

## Methods

A hospital-based observational study was conducted from April 2016 to May 2017at Asella teaching and referral hospital (ATRH) being a federal referral hospital located in Arsi zone, Oromia region, south east Ethiopia. The neonatal intensive care unit (NICU) is one of the wards with the highest performance of the hospital with the admission of close to 1200 neonates per year. Microbiology, hematology and biochemistry diagnostic laboratory services are available around the clock.

Sample size was calculated based on prevalence reports of neonatal sepsis from different studies ranging from 20 to 40% [1, 8–10]. By applying single population proportion and allowing 5% margin of error (d), with 95% CI (zα/2 = 1.96); n = (Za/2)^2^ P(1-P)/d^2^ = (1.96)^2^ 0.3(0.7/ (0.05)^2^ = 322.

During the study period a total of 901 neonates were admitted to NICU from which the study participants were selected based on WHO neonatal sepsis screening tool augmented with national Neonatal Intensive Care Unit Manual [5, 7]. Neonates meeting the inclusion criteria but being too critically ill to undergo the necessary laboratory evaluation and other procedures were excluded. Accordingly, data of 19 neonates were excluded and a total 303 study subjects were analyzed.

### Operational definition

Early onset sepsis (EONS): Sepsis diagnosed in the first six days of life [5, 7].

Late onset sepsis (LONS): Sepsis diagnosed between age of 7 to 28 days of life [5, 7].

Premature rupture of membrane (PROM)**:** rupture of membrane before onset of labor [5, 7].

Prolonged premature rupture of membrane (PROM)**:** PROM lasting for more than 18 h [5, 7].

Prolonged labor**:** Total duration of labor for more than 24 h [5, 7].

Low Apgar Score: Apgar score less than seven [5, 7, 11].

First-line antibiotics: Ampicillin and Gentamycin [5, 7, 11].

Second-line antibiotics: Third generation Cephalosporins [5, 7, 11].

Third-line antibiotics: Vancomycin, Amikacin, and Ciprofloxacin [5, 7, 11].

A standardized questionnaire was prepared by reviewing relevant literatures and neonatology text books and translated to local Amharic language to capture demographic data, risk factors and clinical features of sepsis. After the neonates were enrolled in the study, the mothers were interviewed at a convenient, comfortable and confidential area.

Blood cultures are the gold standard test for the diagnosis of blood stream infection and should be performed in all cases of suspected sepsis prior to administering antibiotics. Accordingly, blood cultures were taken of neonates with clinical diagnosis of. Under preferably aseptic techniques a blood sample was collected by trained laboratory technicians: The laboratory technicians wore sterile gloves during the procedure and prepared a patch of skin approximately 5 cm in diameter over the proposed site of veni-puncture. This area was cleansed thoroughly with 70% isopropyl alcohol, followed by povidone-iodine, and followed again by alcohol. The skin was allowed to dry for at least 1 min before veni-puncture. One-mL sample of blood was drawn from a fresh veni-puncture site and added to a bottle containing 5–10 mL of blood Agar culture media. The blood cultures were incubated aerobically at 37 °C and observed daily for consecutive three days for preliminary results by checking the presence of one of the following findings on culture media: hemolysis, air bubbles (gas production), and coagulation of broth [12]. At the same time, subcultures were made during three successive days on enriched and selective media including blood agar, chocolate agar, MacConkey agar and mannitol salt agar plates and examined for growth after 24–48 h of incubation. Showing no growth on the 7th day, blood cultures were reported as sterile. Isolated bacteria were identified using different standard techniques like Gram stain reaction, biochemical reaction properties (Lactase, Catalase, Indolase), morphological and colony characteristics [12]. Antimicrobial sensitivity testing was performed by Kirby Bauer diffusion method using Mueller Hinton agar with incubation of 24 h at 37 °C. according to Clinical Laboratory Standard Institute standards (CLSI) [8].

Testing was done for antibiotics used for first-, second- and third-line treatment in neonatal sepsis at ATRH [8]. The following antibiotic discs were used: Ampicillin (10 μg), Cloxacillin (5 μg), Gentamicin (10 μg), Amikacin (30 μg), Chloramphenicol (30 μg), Ceftriaxone (30 μg), Ciprofloxacin (5 μg), Vancomycin (30  μg), Clindamycin (2 μg) and Erythromycin (15  μg).

Data was entered into EPI-INFO version 3.5.1 for cleanup and anthropometric interpretation; and then, data were exported to SPSS version 21 edition for further analysis. Frequencies, proportion and summary statistics were used to describe the study population in relation to relevant variables. *P*-values < 0.05 were considered statistically significant.

## Results

A total of 303 (33.6%) neonates were admitted with the diagnosis of clinical sepsis of which 88 (29.4%) were culture proven. From those with culture confirmed neonatal sepsis, male constituted for 52 (59.1%) while females were 36 (40.9%). When looking at bacteria isolates disaggregated to the age of neonates at presentation, 37 (42.4%) bacteria isolates were identified from EONS while 54 (61.2%) from LONS which is statistically significant (*p* = 0.001). neonates born to mother who took antibiotic during labor and delivery were having two-fold reduced risk of acquiring EONS compared to LONS [AOR 2.02 (95% CI 1.17–3.50, *p* = 0.011]. Eight (9%) neonates with culture proven sepsis were born at home while 80 (91%) were born at a health institution (health center or hospital). Babies with low 5th minute Apgar score had high associated with culture confirmed neonatal sepsis [AOR 2.10 (95% CI 1.18–3.73, *p* = 0.001].

### Patterns of isolated organism

Gram-positive bacteria accounted for 49 (55.7%) while the remaining 39 (44.3%) were Gram-negative bacteria. Most Gram-positive [33 (67.3%)] bacteria were reported from neonates with clinical diagnosis of LONS at the time of presentation (*p* = 0.001). About 23 (63.3%) of bacterial isolates from EONS were Gram-negative while close to two-third of identified bacteria from LONS were Gram-positive (Table 1). When disaggregating to specific bacteria pathogen; CoNS (22,25%) and E. coli (18,20.5%) were by far the leading causes of neonatal sepsis in our study.

**Table 1:** Cross tabulation showing distribution of isolated bacteria based on the age at time of sepsis diagnosis, NICU, ATRH, May 2016–April 2017

Isolated bacteria	EONS, n (%)	LONS, n (%)	*p* value
*CoNS1*	5 (22.7)	17 (77.3)	0.001
*E. coli*	12 (66.7)	6 (33.3)	
*S. aureus2*	3 (18.7)	13 (81.3)	
*Klebsiella spp.*	4 (36.4)	7 (63.6)	
*Enterobacter spp.*	2 (28.6)	5 (71.4)	
*Enterococcus spp.*	3 (50)	3 (50)	
*Citrobacter spp.*	1 (33.3)	2 (66.7)	
*others* ^a^	3 (60)	2 (40)	

CoNS1, Coagulase negative staphylococcus S. aureus2, staphylococcus aureus

^a^ Include: Streptococcus pneumoniae, Listeria monocytogenes and candida

### Sensitivity patterns of isolated bacteria

#### Gram-positive bacteria

Most Gram-positive bacteria isolates were from LONS, possibly being hospital acquired infections. These bacteria were highly resistant to first-line and second line antibiotics (Ampicillin and Gentamycin) and third generation cephalosporins used at NICU. The resistance rates of CoNS, S. aureus and Enterococcus against Ampicillin were 20(91%), 11(69%) and 2(33.3%) respectively (Table 3). Similarly, the resistance rates of these three organisms to Gentamycin were 14(63.6%), 9(56.6%) and 4(66.7%) respectively. Identified Gram positive bacteria were also highly resistant to third generation cephalosporins with a cumulative resistance rate against Ceftriaxone, Ceftazidime and Cefotaxime being 29(60%), 23(47%), and 31(64%) respectively.

Isolated Gram-positive bacteria showed better susceptibility patterns for Vancomycin, Clindamycin, Ciprofloxacin and Chloramphenicol (Table 2). Significant methicillin resistance rate was detected in *Staphylococcus aureus* and CoNS which were 11 (69%) and 22 (100%) respectively.

**Table 2:** Antimicrobial resistance patterns of isolated Gram-positive bacteria at NICU; ATRH. April 2016–May 2017

	CoNS	S. aureus	Enterococcus spp.
	n (%)	n (%)	n (%)
Ampicillin	20 (91)	11 (69)	2 (33.3)
Gentamycin	14 (63.6)	9 (56)	4 (66.7)
Ceftriaxone	16 (73)	9 (56)	3 (50)
Ciprofloxacin	8 (36.2)	4 (25)	1 (16.7)
Cotrimoxazole	16 (73)	11 (69)	4 (66.7)
Vancomycin	6 (27.3)	3 (19)	1 (16.7)
Chloramphenicol	10 (45.5)	6 (37)	2 (33.3)
Clindamycin	4 (18)	3 (12)	2 (33.3)
Erythromycin	15 (68)	9 (56)	5 (66.7)
Cloxacillin	22 (100)	11 (69)	NT^a^

^a^NT, not tested

#### Gram-negative bacteria

In the current study, isolated Gram-negative bacteria were also highly resistant to commonly used empiric antibiotics at our NICU (Table 3). E. coli and Klebsiella species were extremely resistant to Ampicillin [12(66.7%) and 10(91%) respectively]. Similarly, these bacteria were also highly resistant against Gentamycin [11(55.6%) and 9(82%) respectively].

**Table 3:** Antimicrobial resistance patterns against selected gram-negative bacteria; at NICU, ATRH. April 2016–May 2017

	E.coli	Klebsiella spp	Citrobacter	Enterobacter spp
	n(%)	n(%)	n(%)	n(%)
Ampicillin	12 (66.7)	10 (91)	NT^a^	6 (85.5)
Gentamicin	10 (55.6)	9 (82)	2 (66.7)	6 (85.7)
Cefotaxime	11 (61.1)	9 (82)	1 (33.3)	3 (43)
Ciprofloxacin	5 (22.3)	3 (27)	1 (33.3)	2 (28.6)
Cotrimoxazole	11 (63.6)	8 (73)	NT^a^	5 (71.4)
Chloramphenicol	8 (50)	6 (56)	NT^a^	NT^a^
Amikacin	4 (22.2)	4 (36)	NT^a^	3 (43)
Erythromycin	12 (66.7)	9 (72)	NT^a^	NT^a^

^a^ Not tested

*E. coli* and *Klebsiella* resistance rates against Cefotaxime, one of the commonly used third- generation Cephalosporin at our NICU were also high. Chloramphenicol, Ciprofloxacin and Amikacin showed more effectiveness against identified Gram-negative bacteria.

### Multidrug-resistant (MDR) bacterial isolates

Most bacterial isolates from blood culture were found to be multidrug-resistant, mainly against first- and second-line antibiotics. About two-third of *E. coli*, 10(91%) of *Klebsiella spp.*, 6(85.7%) of *Enterobacter spp* and 3(50%) of *Enterococcus spp.* were reported to be MDR.

## Discussion

In the current study, about one third of the total neonatal admissions were due to clinical sepsis with or without bacterial growth in blood cultures still remains the most important cause of neonatal morbidity. This finding is consistent with reports from other developing countries [1–3]. The rate of blood culture confirmed neonatal sepsis were significantly lower among neonates with EONS than neonates with LONS. One possible explanation for the difference in blood culture results could be because of the routine utilization of antibiotics during obstetric care which might affect the blood culture yield of the neonates as there is significant transplacental transfer of these antibiotics to the fetus. Gram-positive bacteria were the most commonly isolated organisms causing neonatal sepsis in this finding which is in congruent  with study reports from Egypt, Uganda and other developing countries [3, 4, 9, 13, 14]. Babies with low 5^th^ minutes Apgar score had high risk of developing culture-confirmed Gram-positive neonatal sepsis which could be explained by the fact that most neonates with low Apgar score might undergo extensive manipulation and resuscitation predisposing them for possible invasive colonization with Gram-positive bacteria. Also, this finding is in accordance with studies from Ethiopia and Tanzania [1, 2]. The majority of the isolates were found in LONS, being previously described in a study from Tanzania- Muhimbili which reported S. aureus as the leading cause of neonatal sepsis [1, 15]. Similar findings were reported from Egypt, Tanzania, Uganda, Ethiopia and other developing countries which showed Gram-positive bacteria as predominant isolates causing neonatal sepsis [6, 7, 11, 16–18].

Guidelines on neonatal sepsis management in most centers [7, 19] recommend Ampicillin and Gentamycin as first-line empiric therapy. Unfortunately, most identified bacteria were highly resistant in this current report. Egypt and India [6, 7] showed high resistance rates of isolated bacteria against Ampicillin (85–95%) and Gentamycin (57.3–72%). The demonstrated high rate of antimicrobial resistance (AMR) could be indicating overutilization of the named drugs as empiric treatment for most other common neonatal problems which were not actually infectious in origin. Additionally, most neonates with culture proven bacteremia were born at a health institution where most neonatal sepsis is arising from hospital acquired infections. Resistance rates of isolated Gram-positive bacteria against third generation Cephalosporines were also high in our study which is consistent with studies from Nigeria, Tanzania, Georgia, Iran and other developing countries [11, 12, 15, 17, 19–23].

Methicillin resistant S. aureus (MRSA) and MDR against both, Gram-positive and Gram-negative bacteria isolates were very high in our study finding which is in agreement with most studies [1, 2, 17, 18, 24].

Our study demonstrated a better  susceptibility of isolated Gram-positive bacteria against Vancomycin, Clindamycin and Ciprofloxacin, which is also supported by the study from India and other reports [1–4, 9, 13, 15, 17, 24]. This could be explained by less utilization of these antibiotics for two reasons: First, the antibiotics are used as third line options indicating less utilizations of these agents at NICU. Second, Ciprofloxacin is not validated to use among younger children unless benefit-risk analysis warrants its utilization and, Amikacin is not easily accessible in most centers showing its lower rate of utilization making most isolated bacteria better susceptible to these two antibiotics. Still about one-fifth of isolated S. aureus were found to be resistant for vancomycin which is in contrary with study findings from Vietnam and Egypt showed no resistance strains of S. aureus against Vancomycin [7, 21]. This could be explained by increasing trends of utilization of this antibiotic as third line because most first-line and second-line antimicrobial agents have been failing which is in line to other study findings [5, 10, 11, 21, 22, 25–27], *E. coli* and *Klebsiella spp.* the two predominant Gram-negative bacteria isolates in our study, were highly resistant to Ampicillin, Gentamycin and third generation Cephalosporines. Better susceptibility of *E. coli* and *Klebsiella spp.* for Ciprofloxacin and Amikacin were reported in our study which have been also  demonstrated in other study findings [11, 16]. These two drugs could be a potential antibiotic of choice for empiric treatment of neonatal sepsis in the future.

### Limitations of the study

Some of the study participants were referred to our hospital and might have been partially treated or their mothers might have received antibiotics during labor and delivery which could significantly affect the yield of blood culture. There was no consideration of isolating anaerobic bacteria, which may cause neonatal sepsis rarely [6, 7, 24, 28]. CoNS isolated in early neonatal sepsis may be due to contamination during blood collection. Extended beta lactamase resistant strains were not determined since there was limited availability of biochemical tests (e.g. IMVIC, MR-VP, different sugar tests, KIA) to solidify the research output and furthermore, molecular methods were not available.

## Conclusion

CoNS, *S. aureus*, *E. coli* and *Klebsiella spp.* were the leading causes of neonatal sepsis in our study finding. These bacteria isolates were highly resistant to first- and second-line empiric antimicrobials used at NICU contracting antimicrobial choices for management of neonatal sepsis. Third line antibiotics relatively effective against isolated bacteria. High utilization rate of antibiotics is the most important contributory factor for the development of AMR and continuous surveillance is needed in order to keep national guidelines on antimicrobial therapy updated.

## Abbreviations

AMR: Antimicrobial resistance
ATRH: Asella Teaching and Referral Hospital
CONS: Coagulase negative staphylococcus aureus
CPAP: Continuous positive airway pressure
EONS: Early onset neonatal sepsis
LBW: Low birth weight
LONS: Late onset neonatal sepsis
LP: Lumber puncture
MDR: Multidrug-resistant
MRSA: Methicillin resistance S. aureus
NCCLS: National Committee for Clinical Laboratory Standards
NICU: Neonatal intensive care unit
S. aureus: Staphylococcus aureus
SVD: Spontaneous vaginal delivery
WBC: White blood cell count
WHO: World health organization
